# Buying Years to Extinction: Is Compensatory Mitigation for Marine Bycatch a Sufficient Conservation Measure for Long-Lived Seabirds?

**DOI:** 10.1371/journal.pone.0004826

**Published:** 2009-03-12

**Authors:** José Manuel Igual, Giacomo Tavecchia, Stephanie Jenouvrier, Manuela G. Forero, Daniel Oro

**Affiliations:** 1 Imedea-CSIC/UIB, Esporles, Spain; 2 Estacion Biologica de Doñana-CSIC, Department of Biological Conservation, Sevilla, Spain; 3 Woods Hole Oceanographic Institution, Woods Hole, Massachusetts, United States of America; University of Bristol, United Kingdom

## Abstract

Along the lines of the ‘polluter pays principle’, it has recently been proposed that the local long-line fishing industry should fund eradication of terrestrial predators at seabird breeding colonies, as a compensatory measure for the bycatch caused by the fishing activity. The measure is economically sound, but a quantitative and reliable test of its biological efficacy has never been conducted. Here, we investigated the demographic consequences of predator eradication for Cory's shearwater *Calonectris diomedea*, breeding in the Mediterranean, using a population model that integrates demographic rates estimated from individual life-history information with experimental measures of predation and habitat structure. We found that similar values of population growth rate can be obtained by different combinations of habitat characteristics, predator abundance and adult mortality, which explains the persistence of shearwater colonies in islands with introduced predators. Even so, given the empirically obtained values of survival, all combinations of predator abundance and habitat characteristics projected a decline in shearwater numbers. Perturbation analyses indicated that the value and the sensitivity of shearwater population growth rates were affected by all covariates considered and their interactions. A decrease in rat abundance delivered only a small increase in the population growth rate, whereas a change in adult survival (a parameter independent of rat abundance) had the strongest impact on population dynamics. When adult survival is low, rat eradication would allow us to “buy” years before extinction but does not reverse the process. Rat eradication can therefore be seen as an emergency measure if threats on adult survival are eliminated in the medium-term period. For species with low fecundity and long life expectancy, our results suggest that rat control campaigns are not a sufficient, self-standing measure to compensate the biological toll of long-line fisheries.

## Introduction

Human activities alter ecosystem functioning by modifying the physical characteristics of the environment and the connections within biological networks. For unavoidable impacts, environmental agencies have proposed compensatory mitigation measures that are not intended to restore the original state of the system, but rather act to compensate for the generated loss by enhancing its global functioning [1]. The concept of compensatory mitigation was originally introduced for the restoration and maintenance of natural habitats along the line of the ‘polluter pays principle’ or ‘extent polluter responsibility’, adopted by the EU in the early 1970s to regulate environmental damages [2]. The social importance of fisheries, their economic value [3] and their biological toll, engender apprehension about the sustainability of the exploitation of marine resources. As a response to this concern, a compensatory measure has been recently proposed to compensate for the impact of fisheries on marine top-predator populations [4]. For example, increased adult mortality due to bycatch of adult birds in long-line fisheries has caused many seabird populations to decline [5]–[7]. Among top marine predators, seabirds are unusual because they have to reproduce on land, where additional threats, linked again to human activities, may further jeopardise their populations. Terrestrial predators, e.g. rats *Rattus* spp., introduced by humans in historically predator-free islands actually are an additional driver for population extinction [8], [9]. Wilcox and Donlan (2007) proposed a compensatory action based on the eradication of ship rats *Rattus rattus* to be funded by the local long-line fishing industry. The compensatory efficiency of this measure however has been recently questioned as *i*) the measure can only be useful to small species with terrestrial threats (*i.e.* seabirds) and *ii*) the negative population trend is likely to continue even after rat eradication [9]–[11]. Also, cases of long-term coexistence of rats and seabirds indicate that the compensatory action might not always be justified [12]–[14]. Despite the growing debate on rat control as a compensatory measure, there is a lack of knowledge on the actual demographic consequences of rats on the population dynamics of seabirds. Rats may prey on small adult seabirds, e.g. the European Storm petrel, *Hydrobates pelagicus* [15], [16], but their most documented impact is on breeding success [17]–[19]. This impact is especially prevalent in medium-size species with low behavioural plasticity such as most Procellariiformes (petrels, shearwater and albatrosses) that exhibit high site fidelity, lay a single egg and do not have replacement clutches [20]. The positive relationship between rat control efforts and seabird breeding success [13], [21], [22] is frequently used as justification of rat control or eradication campaigns; however in some cases, the impact of rats on seabird extinction probability is controversial [23] . Thus, the question of the efficiency of compensatory measures is still open. Part of the problem is the difficulty of obtaining robust estimates of seabird demographic parameters, *i.e.* survival and reproductive success, and of measuring their association with rat abundance. Here, we present a model for a population of Cory's shearwaters *Calonectris diomedea* that integrates experimental measures of predation by ship rats and island habitat structure. Adult survival was expressed as a function of a hazard rate that represents a hypothetical additional adult mortality, *i.e.* long-line bycatch. We investigated the demographic influence of these factors on population growth rate using sensitivity analysis and evaluated the efficiency of compensatory mitigation measures.

## Results

### Rat abundance, habitat structure and shearwater demographic parameters

The index of rat abundance, *R*, was negatively correlated with rat control effort in the previous campaign, *E* (*R* = 20.01−0.09*E*, R^2^ = 0.89; P<0.001). Eradication of rats occurred in 2005 since neither captures of rats nor signs of rat presence or predation were recorded in the following two years. About two-third (62.85%) of the variability in nest habitat characteristic was explained by the 1^st^ component of the CatPCA (eigenvalue for dimension one = 5.03, Cronbach's α = 0.92). The index achieved high values for nest with low vegetation cover, high ground complexity, high burrow density and a more central position within the colony. Nests with higher index of habitat structure were less accessible to rats.

Shearwater breeding success was negatively associated with rat abundance (model 1, Table 1) and the strength of this relationship was influenced by nest habitat structure as a result of the interaction between rat abundance and nest characteristics. The fit of this model was checked by inspecting the distribution of residuals (Pearson's normality test *P* = 11.3663, *p* = 0.3297). Similar results were obtained when the random effect was not considered (results not shown). No further simplifications of the model were possible because the interaction term, *R*×*H*, was significant (*Z* = 3.78, *p*<0.001). We built a particular model that included the effect of rat density, *R*, and the statistical interaction between rat abundance and habitat, *R*×*H*, but not the main effect of habitat, *H* (model 2 in Table 1). This model assumes the same breeding success regardless of the complexity of the habitat in the absence of rats and must be viewed as more realistic than model 1 (Table 1). We found that local adult survival was constant over time (0.867, 95% CI: 0.834–0.894) and independent from rat abundance (*F*_1,7_ = 0.638, *p* = 0.448).

**Table 1:** Modelling shearwater breeding success.

Model	Notation	Estimates	se	*Z*	*p*
*1*	*Intercept*	0.668	0.147	4.544	<0.001
	*R*	−0.651	0.090	−7.225	<0.001
	*H*	0.4182	0.139	2.999	<0.01
	*R*×*H*	0.3710	0.098	3.779	<0.001
*2*	*Intercept*	0.661	0.147	4.495	<0.001
	*R*	−0.653	0.090	−7.232	<0.001
	*R*×*H*	0.405	0.083	4.886	<0.001

The effect of rat abundance (R), habitat structure (H) and their statistical interaction (R×H) on shearwater breeding success has been modelled through logistic regressions using the breeding output of 101 nests monitored from 1997 to 2007. The nest identity was taken as a random effect to account for multiple entries from the same nest.

### Rat, habitat, hazard rate and population growth

**Figure 1:**
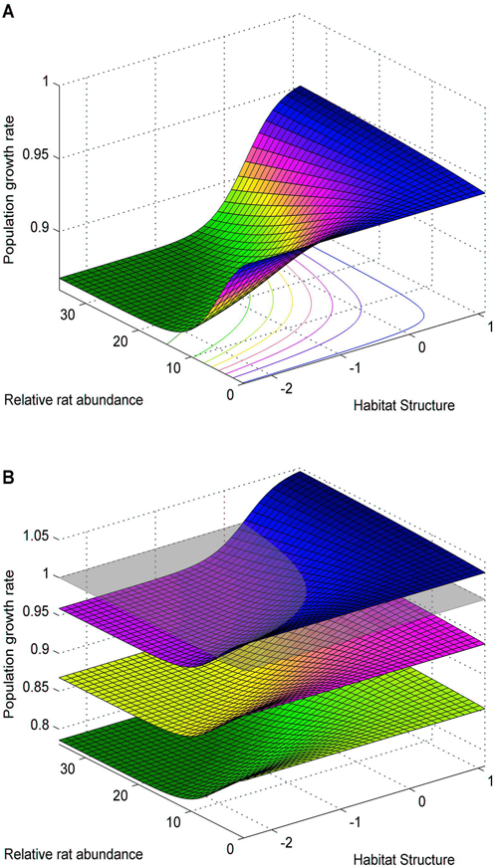
Growth rate, rats and habitat structure. Population growth rate, λ, as a function of rat density and habitat structure. a) Adult survival is 0.87 as estimated at Chafarinas Islands. b) Changes in λ in relation to increasing harvesting rate, z; surfaces from bottom to top correspond to z = 0.1, 0 and −0.1, respectively. The grey surface indicates population stability. In all scenarios, different combination of rat density and habitat structure result in similar values of λ.

**Figure 2:**
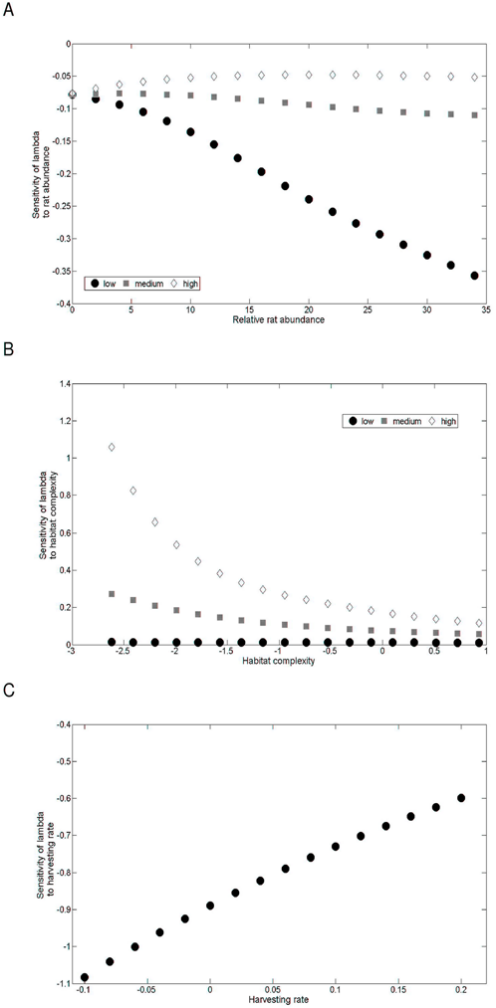
Sensitivity analysis. Sensitivity of lambda to (A) rat abundance for high (◊), medium (▪) and low (•) habitat structure, (B) habitat structure for high (◊), medium (▪) and low (•) rat density, and (C) to hazard rate with habitat structure and rat density set to their average levels. In all graphs survival is set to the value estimated from individual encounter histories (0.87). A change in hazard rate shows by far the highest sensitivity than a change in rat density and habitat structure.

Using the estimated demographic parameters for the average rat abundance and habitat structure, the population growth rate of the deterministic model was 0.934 (95% CI: 0.888–0.981). The population is thus projected to decline by 6.6% per year. This projection is in agreement with the negative trend over time in occupation rate of monitored nests observed on Chafarinas (J.M. Igual *unpublished data*). None of the combinations of rat abundance and habitat characteristics predicted an increasing or a stable population (*i.e.* growth rate ≥1) under the current estimate of adult survival (max value of *λ*: 0.941, Fig. 1a). Stable or growing populations were only predicted for higher values of adult survival (Fig. 1b). The population growth rate was more affected by a change in adult survival than by any other parameters (Table 2). As expected the population growth rate was negatively affected by rat abundance, *i.e.* negative sensitivities (Fig. 2). The sensitivity of *λ* to rat abundance is lower at high values of the habitat index. As a consequence a change in rat abundance will have more effect in islands or parts of the island where habitat structure is low. The sensitivity of *λ* to habitat structure is positive, *i.e.* habitat structure positively affects population growth rate. However, it is almost null when rat density is low as a direct effect of the interaction between rat and habitat structure. The sensitivities of the population growth rate to the hazard rate are negative, *i.e.* the population is negatively affected by an additional adult mortality. The deterministic population growth rate calculated by the model using the observed value of *S*, the average habitat structure and the minimum yearly reproductive output (0.28, recorded in 1999) projected a population decline of about 10% per year. Under these circumstances, a population of 1000 pairs with no immigration will decline to 10 pairs in *c.* 50 years. The eradication of rat will have the effect of ‘adding’ *c.* 30 years to this projection, but will not change the fate of the population. Our deterministic model predicts a stable population (*λ* = 1) when *S* is 0.93, corresponding to an increase of about 6% in adult survival when compared to the observed value at Chafarinas Islands.

**Table 2:** Sensitivity and elasticity of the population growth rate.

Demographic parameter	Notation	Value	s.e.	Sensitivity	Elasticity
*Average fecundity*	*F*	0.659	0.010	0.126	0.023
*First-year survival*	*S_0_*	0.520	0.062	0.159	0.029
*Survival from age 1 to age 2*	*S_1_*	0.868	0.013	0.048	0.044
*Survival from age 2 to age 3*	*S_2_*	0.868	0.013	0.048	0.044
*Survival from age 3 to age 4*	*S_3_*	0.868	0.013	0.048	0.044
*Survival from age 4 to age 5*	*S_4_*	0.868	0.013	0.047	0.039
*Survival from age 5 to age 6*	*S_5_*	0.868	0.013	0.044	0.031
*Survival from age 6 to age 7*	*S_6_*	0.868	0.013	0.191	0.130
*Survival of breeding adults*	*S_Br_*	0.868	0.013	0.499	0.378
*Survival of non breeding adult*	*S_Nb_*	0.868	0.013	0.104	0.049
*Recruitment probability at age 3*	*G_3_*	0.004	0.001	0.111	0.038
*Recruitment probability at age 4*	*G_4_*	0.044	0.009	0.105	0.038
*Recruitment probability at age 5*	*G_5_*	0.126	0.015	0.091	0.036
*Recruitment probability at age 6*	*G_6_*	0.125	0.014	0.403	0.154
*Probability to skip a reproduction*	*1−G_Br_*	0.11	-	0.069	0.326
*Probability to remain non-breeder*	*G_Nb_*	0.59	-	0.016	0.001

Sensitivity and elasticity of λ to the demographic parameters calculated from a model in the absence of rats and with the observed value of survival.

## Discussion

### Rats, fishery and seabird populations

**Figure 3:**
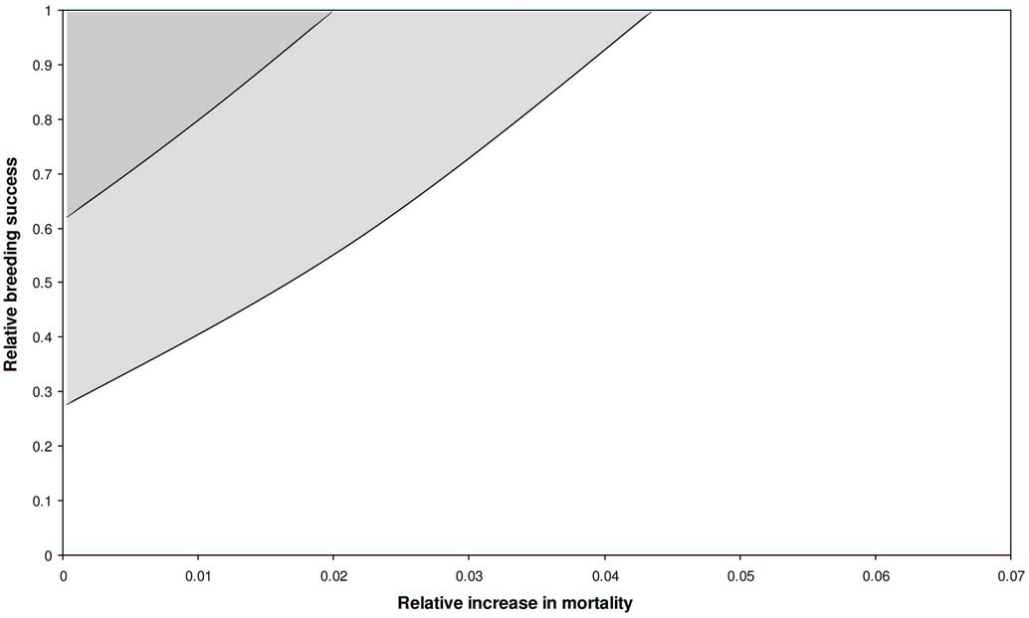
Compensatory breeding success and additional mortality. Relative values of breeding success necessary to maintain a stable or increasing population in relation to an increase in relative mortality (light-gray area: λ≥1, dark-gray area : λ+s.e.≥1). Survival without additional mortality is taken as 0.95 and the average breeding success without rats observed at Chafarinas islands is 0.67 (see text). The deterministic model indicates that a mortality greater than c. 2% cannot be compensated (if λ+s.e≥1 is considered, this threshold value increases to 4.3%).

For the Chafarinas Islands, our deterministic demographic model suggested a decline of the shearwater population under all combinations of habitat structure and rat abundance except in the case when adult mortality and rat abundance are low (Fig. 1). We have shown how the demographic effects of rat predation are mediated by the structure of the habitat. There are important consequences of this interplay between rat and habitat. For example, different combinations of rat density and habitat structure can lead to similar population growth rates. This interaction may explain the persistence of some populations in Mediterranean islets despite ancient rat introduction [12], [14], [16]. We have also shown, using a deterministic model, that when adult survival is low, *e.g.* less than 0.93, rat eradication is not sufficient to reverse a negative trend of the population growth rate. This threshold value is similar to the one found by Mougin et al. [24], [25] during a period of stability of the Cory's Shearwaters at the island of Salvagem Grande (Portugal). The sensitivity of population growth rate to breeding parameters is lower than to adult survival. We can express our results in relative terms using the breeding success in absence of rats (0.67) and assuming an adult survival probability of 0.95 in the absence of additional sources of mortality [24]. Given these values, the maximum increase in mortality probability that can be compensated, *i.e. λ*≥1, by an increased in the breeding success is 2% (Fig. 3). This maximum mortality threshold increases to 6% when one considers *λ*+s.e.≥1 (Fig. 3). A theoretical value of the maximum level of additional mortality can also be estimated using demographic invariants [26] as: Δ*F*/*F*≈*TK* , where Δ*F*/*F* is the relative change in fecundity needed to compensate a change *K* in relative mortality and *T* is the generation time [26]. Given this approximation, in a species with a generation time of *c.* 18 years, as in the Cory's shearwater, a change of 38% in fecundity is needed to compensate a 2% increased in mortality. The maximum change in breeding success observed at Chafarinas Islands was of 40% (average values of breeding success with and without rats were 0.40 and 0.67, respectively). Similarly, Hunter and Caswell [27] showed that bycatch adult mortality has a much greater impact on the population growth rate of Sooty shearwater than did harvest of chicks by Maoris. Cuthbert, Fletcher and Davis [28], also demonstrated that a change of 1% in adult survival had a far greater effect on the distribution of the population growth rate of Hutton's Shearwater *Puffinus huttoni* than increasing fledging success by 5%. Results from the sensitivity analysis of the shearwater population at Chafarinas led to two important conclusions. First, controlling the factors that increase adult mortality, *e.g.* long-line fisheries, has a greater effect on population growth rate than controlling those that limit breeding success, *i.e.* rat density (Table 1). Second, the effect of rats on the population growth rate was modulated by the complexity of the habitat (Fig. 2). When adult mortality is low, reducing rat abundance when nests are accessible, *i.e.* habitat structure is low, has a greater impact on shearwater population growth rate than when habitat structure is high (Fig. 2).

A limitation of our analysis is that the demographic model does not incorporate changes in demographic parameters over time due, for example, to environmental stochasticity. However, environmental stochasticity would cause the long-run growth rate to be lower than the one predicted by a deterministic model [29]. As a consequence, the population growth rates estimated by our analysis have to be considered slightly inflated, a conservative situation when testing the efficiency of mitigation measures.

### Rat eradications and the compensatory mitigation to bycatch

In general terms, rat eradication results in a significant recovery of native biodiversity and is globally acknowledged as a key management option to reduce the impact caused by alien predators [21], [30]. Rat eradication can be successful in isolated and small islets, *i.e.* smaller than 100 ha, while “cost-dependent” [31] or less viable in large islands [30]. Thus rat eradication is an important measure to restore local biodiversity. Wilcox and Donlan (2007) suggested that removal of invasive predators is a more effective measure for seabird conservation from a return-on-investment perspective (*i.e.* percent increase in population growth per dollar invested) and more socio-politically feasible than imposing restriction to fishery. There are two problems with this argument : the first is that the impact of predators depends on their targeted prey (i.e. the impact is much higher when predation is on adults) and the second is the lack of a robust realistic estimation of the demographic consequence of rats and, ultimately, of the effectiveness of the compensatory mitigation measure [9]–[11]. For shearwaters breeding in the Chafarinas Islands, the eradication of rats does not seem a sufficient measure for mitigating additional sources of adult mortality, such as that caused by long-line fisheries. Reducing rat abundance when adult mortality is high will have little effect on the population growth rate, a result practically independent of habitat structure (Fig. 1b and 2). In our study population, eradication increased population growth rate but not enough to reverse a negative trend caused by high adult mortality. In this case, rat control allows to us to “buy” years before extinction but not to reverse the process. However, postponing the extinction of the population a few decades may be important as an emergency measure if threats on adult survival can be eliminated in the medium-term period.

Although our results allow a certain degree of generalization, in particular to long-lived species with low fecundity, they cannot be extended to all seabirds affected by rats or by other introduced predators. The first problem to be considered is the complex interplay between rat predation and habitat structure. A second problem is that, in our system, predation only affected birds' breeding output – a parameter with little impact on the long-term population growth. When predation affects adult birds, it is expected to have a greater impact on seabirds' demography. This is the case for predation by rats on small seabirds (such as storm petrels) or by other introduced carnivores on larger species. In these cases, eradication of introduced predators is crucial for population persistence. Finally, bet-hedging species, such as gulls, with similar survival but higher fertility and a younger age of first reproduction compared to shearwaters, are expected to better respond to rat eradication.

In most cases, the first management action in response to introduced predators is the control or eradication of alien species, even when the demographic consequences of predation are not clear. We showed that the interaction between habitat structure and rat abundance is an important factor that managers should consider in the cost-benefit balance of conservation actions. Most studies on seabirds focus on factors that influence breeding success, typically ignoring other parameters despite these might have a greater influence on the population growth rate. In our case, rat eradication can be an effective management measure when adult survival is high and the combined effect of high rat abundance and low habitat structure makes predation to reach extreme values. On the other hand, if adult survival is high there is not a real need for compensatory measures but rat control can be important as a preventive measure and by itself, to restore the biotic interactions between other island communities, such as plants and insects.

## Materials and Methods

### Study area

We studied the relationship between rat abundance, habitat structure and shearwater demographic parameters on the Chafarinas Islands, an archipelago 4.5 km off the Mediterranean coast of Morocco (Western Mediterranean). Human presence on the archipelago dates from the Neolithic [32] and is concentrated today on a military base and a research station on the island of Isabel II. Rat presence in the archipelago had been documented at least since the end of the 19th century [33] but the species was likely there long before this date as a consequence of human settlement. The breeding colony of Cory's shearwater consists of *c.* 8000–2000 breeding pairs [17], and is located on the largest and most rugged island of Congreso. At present, this is the westernmost known colony of the species in the Mediterranean basin.

### Rat abundance, habitat structure and shearwater demographic parameters

On Congreso island, between 1999 and 2003, we obtained an index of rat abundance, noted *R*, as the number of captures per 100 traps per nights [34]. Trapping occurred in October once birds left the colony [13]. Additionally, a rat control campaign was conducted yearly by placing feeding stations baited with poison, until complete eradication was achieved (where the number of rats per night per trap was equal to zero for two consecutive years). The rat control campaign was conducted soon after the rat abundance survey to modulate the eradication effort accordingly [13]. As a measure of rat control effort, denoted *E*, we took the number of stations multiplied by the days of exposure divided by 100. We measured the linear association between the index of rat abundance, *R*, and the effort of rat control. This relationship was used to estimate the values of *R* in 1997 and 2004, when a measure of the effort was available but the corresponding measures of rat abundance index were missing. This was not possible for 1998 because neither *R* nor *E* were measured.

Rat abundance may not directly correlate with breeding success if nest characteristics make them inaccessible to rats [13]. To obtain a measure of nest habitat structure we measured the habitat, the soil structure and the distance from the nearest neighbour nest, of 101 randomly selected shearwater nests in which output was also subsequently monitored (see below). A Categorical Principal Components Analysis (CatPCA in package SPSS; Rel. 11.0.1. 2001. SPSS Inc., Chicago) was used to reduce the variables considered into a smaller number of components. We retained the component with the highest contribution as an index of nest habitat structure, noted *H*, and used it as a predictor of breeding success (see below).

Fecundity, denoted *F*, was estimated by maximum likelihood from the monitoring of nests for which the habitat index was available. As shearwaters lay a single egg, the breeding outcome has been treated as a binomial variable (1 = success; 0 = failure) and modelled with generalized linear mixed models (GlmmMLpackage [35] in R, www.r-project.org). We considered the relative abundance of rat, *R*, the index of nest habitat structure, *H*, and their statistical interaction, denoted *R*×*H*, as predictors of the breeding outcome, as the correlation between these two variables was not significant (Pearson's product moment, *r* = −0.008, *t*_767_ = −0.236, *p* = 0.813). Nest identity was treated as a random variable to correct for the effect of multiple entries from the same nest. Shearwater annual survival, denoted *φ*, was estimated by maximum likelihood from the observed fate of 354 individually marked adult birds captured and re-observed yearly from 1999 to 2007. The analysis of individual longitudinal data followed standard procedures of capture-recapture modelling [36]. After accounting for departures from a general model [37], we tested whether survival was a function of time and of rat abundance index, *R*. Models were built and compared using program MARK [38]. The significance of *R* was estimated using the ANODEV procedure in program MARK [38].

Adult shearwaters may not return to reproduce when individual or environmental conditions are not suitable for breeding. The exact value of this probability is not easy to estimate from capture-recapture data because the detection probability is confounded by temporal emigration. Therefore, we were able to estimate this probability only conditionally on recapture, which is equivalent to assuming a probability of recapture of 1. As a consequence, a bird known to be alive on a given occasion was assumed to have skipped reproduction if not seen at the colony during the observation period. Estimated in this way, the probability of reproductive skipping was 0.11. Similarly, the probability of breeding after a skipping event was 0.59. These values are consistent with those recently reported for the same species [39]. Finally, as our data did not allow a full description of immature survival and breeding probabilities, we used estimates reported for the same species by Jenouvrier et al. from a colony at Lavezzi island, Corsica [39]. In long-lived species population growth rate is little affected by a change in recruitment parameters [see results ; 40]. Therefore, differences in recruitment processes between Lavezzi and Chafarinas Island should not substantially affect our results.

### Adult mortality and hazard rate

Cory's shearwater die in fishing nets and, especially, in long-lines [41], [42], but this additional mortality is difficult to quantify. For the western Mediterranean, Belda and Sanchez [41] provided an estimate of the number of shearwater caught per 1000 hooks set by bottom long lines boats but this number appeared highly variable [43]. Also, the number of birds caught in long lines cannot be directly related to a measure of mortality probability because the origin of dead birds, and thus the number of birds at risk, is unknown [44]. To investigate the demographic effect of an additional adult mortality due, for example, to bycatch, we used the same approach as in Hunter and Caswell [27]. We expressed bird survival, *S*, as a function of an hazard rate, *z*, so that *S* = φexp(−*z*), with φ the local survival probability estimated from individual encounter histories (see above). Adult survival is equal to φ when *z* = 0, which corresponds to the current level of natural mortality and the current rate of mortality at sea, which is unknown in our analysis. Therefore we assume that positive values of the hazard parameter can be interpreted as an increase in the harvesting rate by fisheries. In this case, the hazard parameter is similar to the harvest parameter used in models for exploited populations [27], [45]. On the contrary, negative values of *z* refer to a scenario in which the current mortality risks decrease. To investigate how results change in relation to a change in mortality risks, we considered a range of adult survival variations between 0.96 and 0.78 using *z* = −0.1, −0.05, 0, 0.05 and 0.1. This range corresponds the one observed across shearwater colonies over the species range [46].

### A population model for the Cory's shearwater

**Figure 4:**
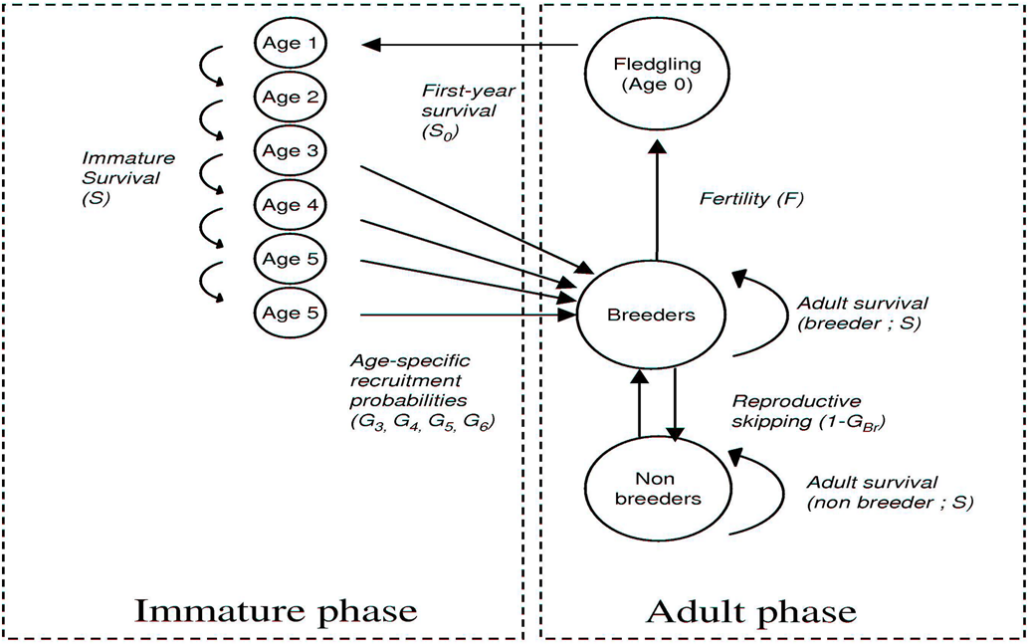
Shearwater life-cycle. We built a population model describing the immature (left) and the adult (right) stages of the Cory's Shearwater life-cycle. Each arrow corresponds to a parameter in eq. 1 describing the transition from the age–stage class *i* to the age-stage class *j* between *t* and *t+1*. The immature part includes the probabilities (G_3_, G_4_, G_5_ and G_6_) that describe the progressive recruitment of immature birds into the breeding pool. Note that adult parameters were assumed to be age-independent.

To explore population trajectories, we combined shearwater survival, fertility, recruitment and breeding probabilities into a stage demographic model [47]. The model is a matrix representation of shearwaters' life cycle with 8 stages according to age and state (breeder-non breeder; Fig. 4). The population matrix **M** (size 8×8) is:
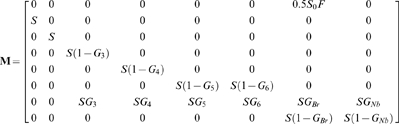
(1)Where:

*S_0_* = is the annual survival from fledgling to the first birth day.
*S* = the annual survival probability expressed as φexp(−*z*), with φ the local survival probability from adult encounter histories and *z* the hazard rate (see above)
*G_j_* = age-specific recruitment probability at age *j*, with *j* = 3,4,5,6.
*F* = the average breeding success modelled as a function of rat density, *R*, and habitat structure, *H*, as 1/(1+exp[−(*α+β*(*R*)*+β*(*R*×*H*))] in which α and *β* are the linear predictors of *F* on a logit scale (see Table 1).
*G_Br_* = the average probability of breeding after a breeding event (here *G_Br_* = 0.89). Note that 1−*G_Br_* is the probability to skip a reproduction after a breeding event.
*G_Nbr_* = the average probability of breeding after a non-breeding event (here *G_Nbr_* = 0.59).

The eight rows of **M** contain the demographic parameters corresponding to the eight age-by-breeding stages, which are from the top to bottom, fledgling, immature from age 1 to age ≥5 years old, adult breeders and adult non-breeders. Each non-zero element in eq.1 corresponds to an arrow in Fig. 4. Thus, the first row contains the parameters related to fertility, *F*, and the first-year survival, *S_0_*, while the rest of the matrix includes the transition probabilities between all stages identified during the time interval *t*, *t*+1. The matrix **M** can be viewed as composed by two sub-matrices corresponding to the two boxes outlined in Fig. 4. The first sub-matrix (size 8×6, left sector of eq.1), describes the immature part of the cycle. It includes a survival parameter, *S*, common to all ages, and the age-specific recruitment probabilities, *G_j_*, expressed as the transition probability at age *j* from the non-breeding to the breeding state. At age 1 all birds are immature and move to the next immature age class with probability *S*. At three years old, immature birds ‘move’ into the adult breeding pool, i.e. row seven, , with probability *G_j_* or move into the next immature age class with probability 1−*G_j_*. The last row of this sub-matrix is empty because immature birds cannot become non-breeding adults. The first row is empty because by definition, immature do not reproduce. The second sub-matrix (size 8×2) refers to the adult phase of the cycle. It is structured into two states, breeder and non breeder, regardless of the age of the bird. As the first sub-matrix, it contains the adult survival parameter, S, and the probabilities, *G_Br_* and *G_Nb_*, to become a breeding after a breeding and a non-breeding event, respectively.

The impact of rats is considered only in breeding birds, whereas the impact of fishery concerns non-breeding and immature birds as well [41]. The population matrix **M** projects the population vector *n* from *t* to *t*+1 as *n_t+1_* = **M** *n_t_*. The asymptotic population growth, λ, is calculated as the dominant eigenvalue of **M**. In a stable population the population growth rate is equal to 1 while lower values indicate a decreasing trend. Finally we used perturbation analyses to investigate how the change on a demographic parameter, a combination of more than one parameters or a covariate, would affect the population growth rate *λ* [48]. The sensitivity of *λ* to a given parameter, *θ*, is a scalar calculated as ∂λ/∂*θ* and gives an indication of how the population growth rate is affected by a change in *θ* [47]. The sensitivity of *λ* to *θ* represents the slope of the relationship between parameter and *λ*, and is positive if population increases when the parameter consider considered increases. The sensitivity to a given covariate, X, affecting *λ* through a parameter *θ*, is calculated as 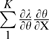, with 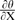 the derivatives of *θ* with respect to the covariate value, and *k* the number of parameters. Sensitivity analyses were computed with program MATLAB [49]. The 95% confidence interval of λ can be calculated combining parameter variances with their respective sensitivity [50].
